# A web-based intervention to support the mental well-being of sexual and gender minoritised adolescents: Formative evaluation of Oneself

**DOI:** 10.1177/20552076251321057

**Published:** 2025-03-13

**Authors:** Mathijs FG Lucassen, Rajvinder Samra, Katherine E Brown, Katharine A Rimes, Alicia Núñez-García, Louise M Wallace

**Affiliations:** 1 School of Health and Psychological Sciences, City St George's, 4895 University of London, London, UK; 2 School of Medicine, 1415 The University of Auckland, Auckland, New Zealand; 3 School of Health, Wellbeing and Social Care, 5488 The Open University, Milton Keynes, UK; 4 School of Life and Medical Sciences, 3769 University of Hertfordshire, Hatfield, UK; 5 School of Mental Health and Psychological Sciences, 4616 King's College London, London, UK; 6 College of Medicine and Veterinary Medicine, 3124 The University of Edinburgh, Edinburgh, UK

**Keywords:** Adolescents, mental health, public health, digital health, LGBTQ+, sexuality, gender minority

## Abstract

**Background:**

Sexual and gender minoritised adolescents are at an increased risk of mental health problems. However, few interventions have been specifically designed to support their mental well-being.

**Objective:**

The purpose of this study was to evaluate Oneself; a prototype bespoke digital mental well-being intervention co-developed with and for sexual and gender minoritised adolescents.

**Methods:**

Think aloud testing of Oneself was conducted with sexual and gender minoritised adolescents. Adult experts appraised Oneself via semi-structured interviews. Additionally, participants completed questionnaires including the System Usability Scale (SUS). Qualitative data were analysed using a general inductive approach.

**Results:**

Participants included 11 sexual and gender minoritised adolescents (aged 14–19, mean 16.3 years) and 14 adult experts (78.6% 30 years or older). Usability, satisfaction and well-being results indicated that Oneself is a promising intervention. The mean SUS score was 78.8% (n = 25), which corresponds to a B+ on the Sauro–Lewis curved grading scale. Six themes were identified across the qualitative data: ‘Clarity and accessibility’ (e.g. refine audio-visual content as well as text), ‘Appeal and depictions’ (e.g. enhance the perspectives and representation), ‘Functionality and development’ (e.g. extend the choice and user options), ‘Safety and privacy’ (e.g. harsh world warnings needed for LGBTQ+ youth), ‘Reaching the end users’ (e.g. promoting Oneself in a youth-friendly way) and ‘Mechanisms of impact’ (e.g. sharing lived experience).

**Conclusion:**

Oneself could be used to help support the mental well-being of users, but modifications are indicated prior to any further testing and consideration of a roll-out.

## Introduction

Sexual and gender minoritised adolescents include all the young people who identify as lesbian, gay, bisexual, transgender/trans, non-binary and queer, as well as other sexual and gender minoritised (i.e. LGBTQ+) youth, such as those who are questioning their sexuality and/or gender. Social progress for sexual and gender minoritised people in high-income Western countries, including the United Kingdom (UK), has advanced in recent decades but sexual and gender minoritised adolescents often encounter mistreatment^1^ and unsupportive environments.^2^ This in turn impacts upon their mental health,^3^ for instance, they are more likely to have depressive symptoms,^4^ self-harm^5^ and experience low subjective well-being^6^ compared to their peers who are heterosexual and cisgender (i.e. a person whose gender identity corresponds to the sex they were assigned at birth). For example, the UK's nationally representative longitudinal Millennium Cohort Study (MCS) of almost 10,000 14-year-olds reported in 2020 that approximately 6% were sexual minoritised (e.g. lesbian, gay and bisexual) adolescents and that these young people had over five times the odds of high depressive symptoms, compared to their heterosexual peers.^6^ Comparatively fewer population-based research studies have been published about transgender and other gender minoritised adolescents. However, results from the MCS found that approximately 1% of adolescents are gender minoritised youth (e.g. transgender or non-binary) and that they are more likely to experience psychological distress than their peers who are cisgender.^7^ For instance, they reported three times the risk of suicide attempts in comparison to cisgender adolescents.^7^ In contrast to many adults, sexual and gender minoritised adolescents cannot simply leave challenging social environments due to certain constraints, such that they are minors who are economically dependent on their families. Further compounding the issues is that sexual and gender minoritised adolescents in Western countries are ‘coming out’ earlier,^8^ when they have had fewer opportunities to develop effective strategies to cope with anti-LGBTQ+ stigma.^9^ Consequently, there is a pressing need for accessible and targeted help to assist these adolescents to develop the psychosocial skills they need to thrive.

Sexual and gender minoritised adolescents have high mental health needs, but despite sexual orientation and gender reassignment being protected characteristics in the UK's Equality Act (2010), few evidence-informed interventions have been developed specifically for them. Earlier systematic reviews of psychosocial treatments identified only a single tested digital tool,^10,11^ Rainbow SPARX (Smart, Positive, Active, Realistic, X-factor thoughts).^12^ This intervention is an adaptation of the main version of SPARX, a form of digital cognitive behavioural therapy in serious game format for the treatment of adolescent depression.^13^ This dearth of research in the field is surprising, considering that internet-based mental health tools have been highlighted as an important way of potentially improving access to evidence-based mental health interventions.^14^ SPARX and Rainbow SPARX were developed in New Zealand,^12^ hence in follow-up research, we sought the views of LGBTQ+ adolescents in England, to determine Rainbow SPARX's suitability within UK settings.^15^ Feedback from sexual and gender minoritised adolescents in that study reinforced a bespoke intervention would be required, rather than amending Rainbow SPARX.^15^ It was recommended that this new resource needed to be cognisant of an intersectionality-informed modern British context.^15^ These views echo the findings from a systematic review which highlighted the importance of certain features for digital health interventions for LGBTQ+ populations, namely that content should be tailored to LGBTQ+ experiences, provide connection to LGBTQ+ communities and link to other relevant LGBTQ+ resources.^16^ A recent review of supports for sexual and gender minoritised adolescents identified 12 psychotherapeutic interventions, five of which were digital.^17^ These primarily addressed depression and anxiety, but none of these interventions have been tested or used in Europe, including within the UK.^17^

Based on the higher risk profile and specific needs of sexual and gender minoritised adolescents, and in line with their preference for a customised digital intervention, we obtained funding from the UK's Medical Research Council to create a co-developed tool. We drew upon aspects of successful youth-focused interventions, such as SPARX, by utilising key evidence-based techniques (e.g. problem-solving skills)^17^ and developed a digital intervention specifically for LGBTQ+ youth. We aimed to create a digital intervention that could support positive skill development for those sexual and gender minoritised adolescents who experience mild mental health problems – adolescents who would not be eligible for state-funded mental health services. The intervention is also for those who do not have mental health problems (e.g. as primary prevention). At the project's outset, we published our study protocol^18^ and since then we have described in detail the co-design processes we employed.^19^ This paper summarises the formative evaluation of this co-developed prototype resource, named Oneself. The research questions guiding the study were:

1. How acceptable is Oneself, in terms of supporting and meeting the needs of sexual and gender minoritised adolescents?
2. In what ways should Oneself be changed prior to being made available to sexual and gender minoritised adolescents?
3. How can web-based resources like Oneself be used in every-day life by sexual and gender minoritised adolescents and other individuals or organisations?

## Methods

This study was a formative evaluation of the Oneself intervention. It was conducted between May and August of 2023. Specifically, think aloud sessions were carried out together with sexual and gender minoritised adolescents between the 5th of May and the 25th of August, whilst interviews with the adult experts were conducted between the 15th of June and the 17th of August. On average, adolescent participants spent almost 2 hours using Oneself during think aloud sessions (mean 111 minutes, range 102 to 122 minutes) and adult interviews lasted on average 39 minutes (range 30 to 57 minutes). In terms of where the study was conducted, adolescent participants could select to have their sessions in-person at their youth centre (all in Greater London or the Home Counties of England) or online via Teams. The adult interviews were completed online using Teams or in-person in London or the Home Counties, depending on the participant's preference.

### Intervention

**Figure 1.**
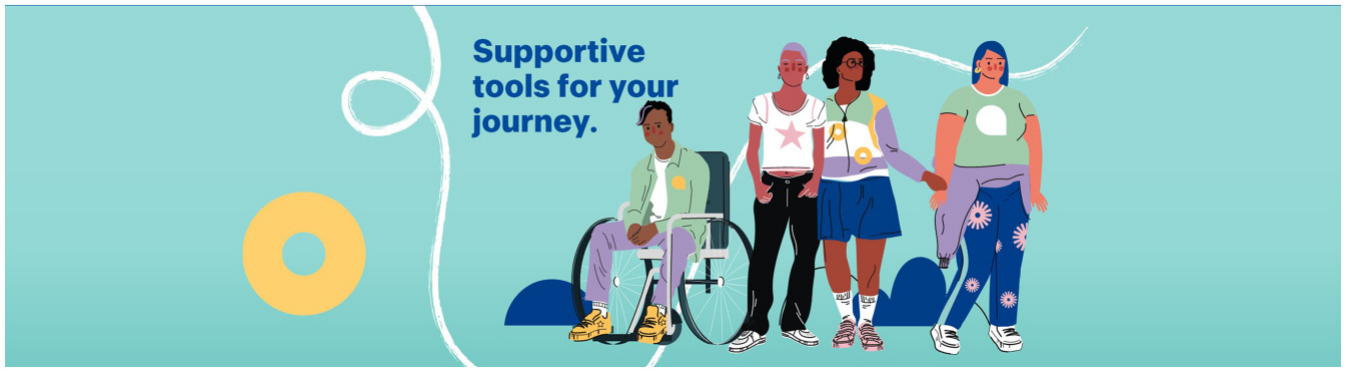
Image from Oneself’s homepage.

The co-development of Oneself has been described in detail previously.^19^ In summary, it features a home/welcome page (see Figure 1), which includes an animation explaining the importance of using a person's correct pronouns (e.g. she/her, he/him and they/them), as well as the opportunity to create a user account and log-in to access further material. There are three main sections of content focused on the priority areas identified by sexual and gender minoritised adolescents, specifically: (a) coming out and doing so safely; (b) managing school environments, including coping with homo-, bi- or trans-phobic bullying; and (c) dealing with parents and families, especially unsupportive family members. Three sexual and gender minoritised contributors (i.e. Chloe, Lilly and Georgie) were identified as part of Oneself's development via specialist media or modelling agencies. They were selected during co-design processes to create video clips for Oneself. Six clips were created that addressed common challenges and strategies for dealing with coming out, school-based experiences and issues to do with parents and families.

**Figure 2.**
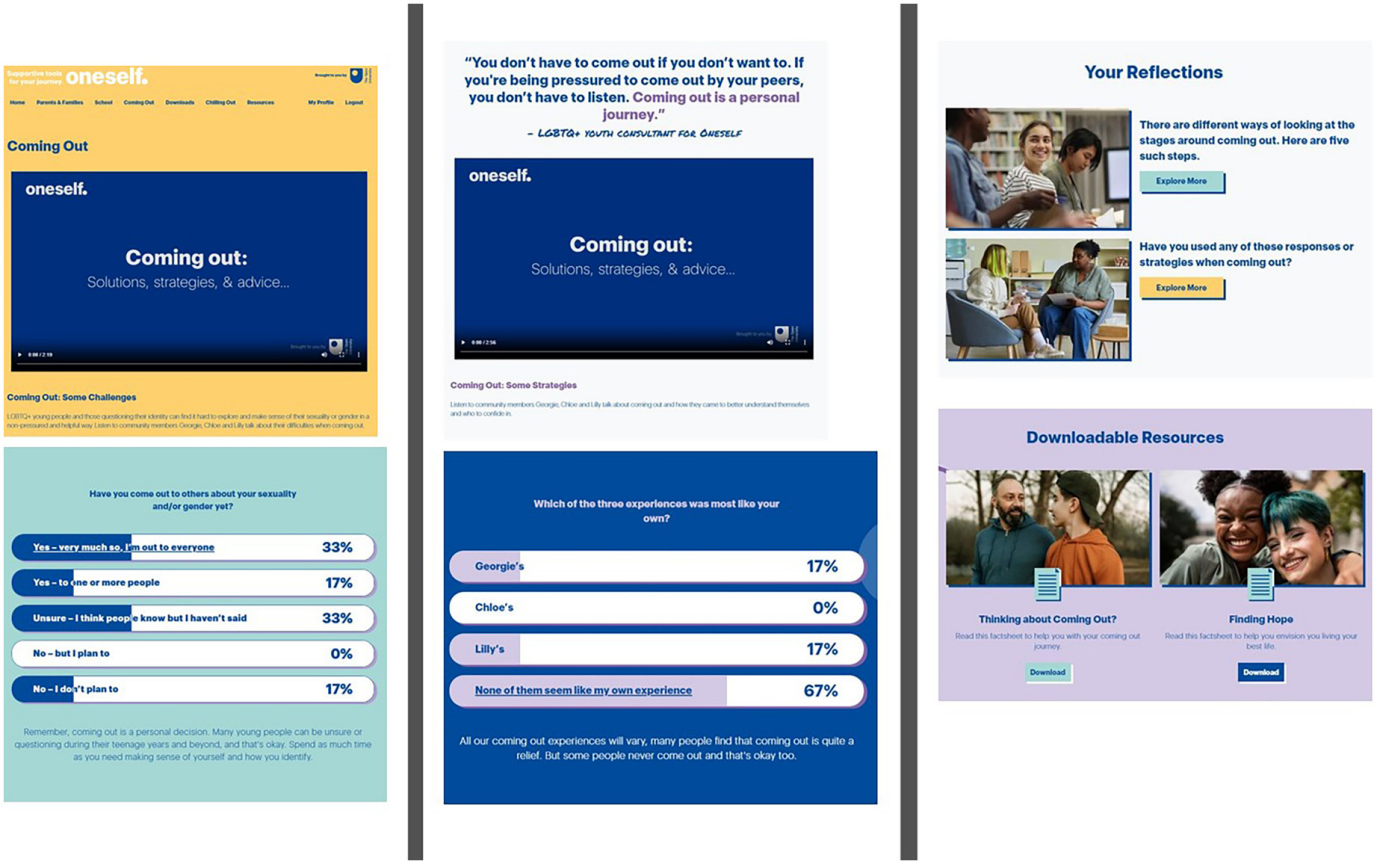
A Oneself topic example (Coming Out).

Oneself draws on therapeutic concepts, such as cognitive reframing, stress reduction and problem-solving techniques. There is also a separate section consisting of audio-recorded relaxation exercises which are delivered by Chloe, Lilly and Georgie; a section with links to other recommended supports and resources; and a downloads section, with further techniques and strategies for enhancing well-being. Techniques designed to foster a sense of community and encourage engagement within Oneself consist of: social polls with Likert-scale responses after each video clip (e.g. ‘Have you struggled with your parents or family being unsupportive of your sexuality and/or gender?’); exercises to help the user reflect on a topic (e.g. an exercise highlighting five main coping strategies to manage school bullying and inviting the user to identify a strategy to try out); and thought-provoking quotes from the sexual and gender minoritised adolescents involved in the co-design of Oneself (e.g. in the dealing with parents and families section – ‘Know that it's a lack of understanding rather than a lack of love’). See Figure 2 for an illustrative example of the Coming Out content.

### Participants

The study was conducted amongst primary and secondary populations. The primary population were the anticipated end users: sexual and gender minoritised adolescents residing in England. We aimed to recruit approximately 10 such adolescents as outlined in our study's protocol,^18^ which aligns with the sample size of prior evaluations of novel digital interventions also using a think aloud approach.^20,21^ The secondary population comprised adult experts, specifically the parents/guardians of sexual and gender minoritised adolescents, youth workers supporting sexual and gender minoritised adolescents and public health service commissioners. We sought the input of these adult experts given their expertise in supporting sexual and gender minoritised adolescents and because their views are important in terms of determining whether an intervention, like Oneself, will be endorsed and potentially provided to adolescents. We estimated recruiting approximately five such adult expert participants,^18^ which is comparable to other evaluations of a digital intervention, where adult experts are included alongside the anticipated end users.^22^

### Ethical considerations

Ethical approval for this study was granted by the Human Research Ethics Committee at The Open University (HREC/4059/Lucassen). All participants provided written informed consent. Adolescents aged <16 years assented and required written parental or guardian consent. All study data have been de-identified. Participants were offered gift vouchers as a token of gratitude for their contributions to this study, adolescents received a £20 (circa US$25) voucher for each think aloud session (i.e. a total of £60/circa US$75 in vouchers), and adult experts were offered a £20 (circa US$25) voucher.

### Recruitment

Adolescents were recruited via youth workers providing community-based groups for sexual and gender minoritised adolescents. The youth workers were known to the adolescent participants, and they were all employees of youth centres funded by LGBTQ+ charities and a county council in Southern England. The youth centres were based in a range of geographic locations in the Southeast, but were primarily in Greater London, where their service users are diverse in terms of their ethnicity, socioeconomic status, sexuality (e.g. lesbian, gay, bisexual or questioning) and gender identity (e.g. transgender and non-binary). The organisations were involved in the Oneself project from its commencement. As stipulated in our study's protocol,^18^ the adolescent participants in this study needed to be young people who had not been previously involved in the co-design work associated with Oneself. Our rationale for this was to gain fresh perspectives and to avoid over burdening the more than 50 young people who had engaged in the various stages of the co-design processes.^19^

The youth worker participants were recruited via the organisations, whilst the public health service commissioners and parents were recruited through the professional networks of the researchers. Parents of children under 16 years who had heard about the study from their child were also recruited by the team.

### Procedures

Informed by a prior evaluation of a web-based intervention conducted with sexual minoritised young men,^23^ we used a think aloud approach.^24^ Due to the preferences of the adolescents and the youth workers involved, the three separate think aloud sessions were conducted weekly after school and scheduled to last up to 40 minutes each. All sessions were facilitated by MFGL, and a youth worker was present for participants < 18 years old. Participants had the option to use Oneself via a study laptop or a mobile phone. All the sessions were audio-recorded and the transcripts, which were checked by MFGL or AN-G, comprised the data for the think aloud sessions.

After reiterating the purpose of the sessions, which was to evaluate this new prototype resource, housekeeping points were highlighted (e.g. there would be three sessions). The think aloud approach was then explained to each participant at the start of their first session and reinforced at various times throughout the sessions. For instance, MFGL asked that they ‘think aloud’ or say what they were thinking, as they explored the intervention. This was to include all their thoughts about Oneself, such as ‘I don’t agree with this part’ or, ‘I really like the way this screen looks here’. It also could include any questions they had, like ‘where would I go from this screen?’ or ‘I’m not sure what this means?’. The aim of this approach, as explained to adolescent participants, was to obtain a detailed account of the user's reactions to Oneself, and to determine whether any parts of Oneself are difficult to understand or needed refining. To support the think aloud sessions, we developed a series of questions. For example, as a warm-up item we asked all young people what they thought Oneself would cover prior to logging on for the first time.

Adult participants were asked to review one of the three areas/content sections or the relaxation section prior to their interview. Interviews were conducted at a time and date convenient to them. Whilst discussing Oneself, the participants had the intervention visible to them on a laptop or another screen. They were then asked a series of questions, for example ‘What word or words would you use to describe Oneself?’ After providing their feedback about the area of Oneself that they reviewed in detail, adult participants were asked some more general questions. These included ‘In terms of your overall impressions, what did you…like most about Oneself?…like least about Oneself?’ They were then asked questions about how Oneself could potentially be used outside of a research context and what needs to happen before Oneself can be released to end users.

### Assessments

Adolescents and adults completed the System Usability Scale (SUS).^25^ It is the most widely used standardised assessment of computer systems.^26^ The SUS consists of 10 items (e.g. ‘I think I would like to use this system frequently’) and scores range from 0% to 100% (the best possible score). The Sauro–Lewis curved grading scale has resulted in score range categories being used for the SUS, and this provides an associated grade and percentile range.^27^ For example, 0.0–51.6% corresponds to an ‘F’ grade (percentile range 0–14) whilst 84.1–100% corresponds to an ‘A+’ grade (percentile range 96–100).^27^ By way of context, in assessing 14 commonly used products, Kortum and Bangor^28^ reported average SUS scores which ranged from 56.5% for Excel to 93.4% for Google search.

Adolescents completed the WHO-5 Wellbeing Index^29^ before and after using Oneself. It is a short, self-administered questionnaire covering five items, related to positive mood, vitality and general interests over the previous two weeks. Each of the five items is rated on a six-point Likert scale from ‘All of the time’ (a score of 5) to ‘At no time’ (a score of 0). For instance, item one is ‘I have felt cheerful and in good spirits’. The raw score (maximum 25) is multiplied by 4 to establish a percentage score (100 = the best imaginable well-being). The index has good psychometric properties,^30^ and a WHO-5 cut-off score of ≤50 is recommended when screening for depression.^30^

Adolescents and adults completed the Oneself Satisfaction Survey, a bespoke 20-item questionnaire, after their final think aloud session or their interview. The survey was an adapted version of a questionnaire used to assess SPARX^13^ and Rainbow SPARX.^12^ It consisted of five items about perceived usefulness (e.g. ‘Overall, how useful was Oneself?’) and six items about perceived likability (e.g. likability of ‘You can learn things from Oneself by yourself at your own pace’), these items were assessed using a 5-point Likert scale. The 11 items rated using Likert scales when combined provided a total satisfaction score, which ranged from 11 to 55, where 55 was the highest level of satisfaction possible. The remaining nine items comprised six closed items (e.g. ‘Do you think Oneself would appeal to LGBTQ+ young people?’ and ‘Would you recommend Oneself to LGBTQ+ young people?’) and three open-ended items (e.g. ‘Any ideas for making it better?’).

### Analysis

The quantitative data from the SUS, WHO-5 Wellbeing Index and the Oneself Satisfaction Survey were analysed using the descriptive functions within SPSS 29.0.2.0. We applied a general inductive approach to analyse qualitative data.^31^ This form of qualitative content analysis seeks to build understandings from observations, and not testing pre-existing hypotheses, theories or models. Instead, a general inductive approach focuses on eliciting the perspectives and views of participants related to focused evaluation questions. As suggested by Thomas,^31^ our aim was to investigate common themes, points of agreement/disagreement and interrelationships between themes and subthemes from the data. Data were read with the research questions in mind. MFGL then read and re-read the data several times and identified lower order units of meaning (i.e. initial codes). These were then refined in an iterative fashion in collaboration with RS and then clustered with like units. These were then reviewed for redundancy and checked against the data to ensure the essence of the units. MGFL and RS developed the initial themes and subthemes, using the codes created, which were checked by all co-authors. Names and definitions of each theme were agreed by all co-authors. Quotes encapsulating the themes were selected and are presented in this paper. NVivo14 software was used to manage the data and support analyses.

## Results

### Participant characteristics

Eleven sexual and gender minoritised adolescents (mean age 16.3 years, range 14–19 years) participated. In terms of gender, where adolescents answered an open-ended item, the responses varied. Five were non-binary, inclusive of the participants who were ‘gender queer’ or ‘genderfluid’ (45.5%). Three were female-identified, specifically the ‘female’, ‘she/they’ and ‘woman’ (27.3%) participants. Two were male-identified, i.e. ‘trans-man’ and ‘trans male/other?’ (18.2%), and one participant was ‘questioning’ their gender (9.1%). Eight (72.7%) stated that their gender was different from that assigned at birth (i.e. they are a gender minoritised adolescent). In terms of their sexuality, four (36.4%) were queer, four (36.4%) were pansexual, with one each being lesbian (9.1%), gay (9.1%) or bisexual (9.1%). Eight participants were White (72.7%), two were Black (18.2%) and one (9.1%) participant was of mixed heritage. All 11 had previously had an experience of feeling down or low for more than a few days in a row.

Fourteen adult participants included nine youth workers, three parents and two public health service commissioners. Eleven (78.6%) were 30 years or older, two (14.3%) were 26–29 years old, and one (7.1%) was under 25 years old. Adults also responded to an open-ended item in relation to their gender. Eight participants (57.1%) were female-identified (e.g. ‘cis female’, ‘woman’ and ‘she/her – cisgender’). Two (14.3%) were male-identified (i.e. ‘Male’ and ‘My sex is male’), and two (14.3%) were non-binary. One (7.1%) adult participant stated ‘transmasc/non-binary’, and one (7.1%) wrote ‘prefer not to say’. More than three-quarters (11 adult participants, 78.6%) identified as a member of the LGBTQ+ communities. Approximately two-thirds (nine adult participants, 64.3%) were White, three (21.4%) were Black, one (7.1%) was Asian, and one (7.1%) was of mixed heritage.

### Quantitative findings

*Usability*: SUS scores for adolescent users (n = 11) ranged between 73% and 98%, with a mean score of 82.5% (standard deviation/SD 6.8). The SUS scores for adult participants (n = 14) ranged between 45% and 95%, with a mean score of 75.9% (SD 12.2). For all participants combined (n = 25), the mean score was 78.8% (SD 10.5), which corresponds to a B+ grade (80–84 percentile range).^27^

*Satisfaction*: In terms of participants’ overall satisfaction with Oneself, the mean adolescent score was 46.5 (range 37 to 52, SD 5.4), whilst the mean adult score was 44.2 (range 32 to 51, SD 4.6) where the maximum score possible was 55. Of the three main sections/areas and the relaxation content, ‘learning about coming out’ was rated as the most useful by both adolescents (i.e. 4.4) and adults (i.e. 4.3) (see Table 1). Most sections received a rating of 4 (i.e. ‘Useful’) or above. ‘It was made together with LGBTQ+ young people’ was rated as the most liked feature of the intervention by adolescents (i.e. 4.9) and adults (i.e. 4.6). Whilst ‘It showed me things I didn’t know’ received the lowest mean score from both adolescents (i.e. 3.9) and adults (i.e. 3.4). Most adolescents (n = 8, 81.8%) and adults (n = 12, 85.7%) thought that Oneself should stay as it is, in relation to how long it takes to complete. All the adolescents and 13 of the adults (92.9%) thought that Oneself would appeal to LGBTQ+ young people. Moreover, all the adolescents and 12 (85.7%) of the adults indicated that they would recommend it to other LGBTQ+ young people.

**Table 1.** Oneself satisfaction survey.

	Adolescents					Adults				
	N	Min^a^	Max	Mean	SD	N	Min^a^	Max	Mean	SD
Usefulness of topics (5 = very useful)										
Overall, how useful was Oneself?	11	2	5	4.4	0.9	14	2	5	4.1	1.0
Learning about parents/families	11	2	5	3.8	0.9	14	2	5	4.1	1.0
Learning about managing bullying	11	2	5	4.3	0.9	14	3	5	3.9	0.6
Learning about coming out	11	4	5	4.4	0.5	14	4	5	4.3	0.5
Learning about chilling out/relaxing	11	2	5	3.8	1.0	14	3	5	4.1	0.6
Likability of features (5 = really liked)										
Can learn things from Oneself by yourself at your own pace	11	4	5	4.6	0.5	14	4	5	4.4	0.5
Oneself has video clips of LGBTQ+ people giving advice	11	3	5	4.4	0.8	14	1	5	4.1	1.0
It showed me things I didn’t know	10^b^	3	5	3.9	0.9	12^b^	2	5	3.4	0.8
It is designed for LGBTQ+ young people	11	4	5	4.7	0.5	14	3	5	4.2	0.7
It was made together with LGBTQ+ young people	11	4	5	4.9	0.3	14	4	5	4.6	0.5
It has a UK look and feel	10^b^	3	5	4.0	0.8	13^b^	3	5	3.6	0.7

^a^ The response options for all items were 1 to 5; the Min (i.e. minimum) is the lowest score provided amongst the participants.

^b^ Participant/s for this item responded, ‘not applicable’.

*Well-being*: Baseline WHO-5 Wellbeing Index scores, from immediately prior to the first think aloud session for adolescent participants (n = 11), ranged from 28% to 84%, with a mean score of 51.6% (SD 17.7), which is above the cut-off for depression screening (i.e. ≤50). Mean well-being scores immediately after the last think aloud session improved slightly to 54.7% (n = 11, range 26% to 76%, SD 18.3).

### Qualitative findings

The analysis of qualitative data, which focused on answering the three research questions, resulted in six themes, with between three and six subthemes per theme. In total, there were 25 subthemes (see Table 2 for an overview).

**Table 2.** Overview of qualitative findings.

Themes and subthemes	Illustrative points and quotes
**Theme 1: Clarity and accessibility**	
Subtheme 1.1 – Language	At times it ‘…*reads a bit difficulty* [difficultly]’ (Youth Participant 6), it felt ‘*very academic*’ (Youth Participant 9) or ‘…*there were parts of it where the language is talking like it's talking to an adult to talk to young people*’ (Commissioner 2).
Subtheme 1.2 – Names and pronouns	Youth workers and youths (not parents or commissioners) recommended Oneself make it clearer who was speaking in audio-visual clips as well as their pronouns: ‘…*the videos needed like labels and pronouns for the people*’ (Youth Participant 2).
Subtheme 1.3 – Refining audio and visual content and text	Certain elements required written text (or more concise text), including audio or visual elements provided alongside the written content: ‘*I think just visuals* [alongside text] *would help*…’ (Youth Participant 1).
Subtheme 1.4 – Accessibility	Oneself should be made more accessible (e.g. to those with dyslexia): ‘[when asked about refining it]:…*I liked least…one of the sections…just the way the graphics had been laid out made it a bit difficult to read*’ (Youth Worker 8).
**Theme 2: Appeal and depictions**	
Subtheme 2.1 – Visually appealing versus distracting or off-putting	‘*I think it looks welcoming*’ (Commissioner 1) and ‘*It looks nice*’ (Youth Participant 7) contrasted with off-putting aspects e.g. animations ‘*popping up*’ (Youth Participant 6) in audio-visual clips.
Subtheme 2.2 – Relatability and realness	Valuable to have real-life people as contributors (i.e. Georgie, Chloe and Lilly), but they did not appear to be school-aged and there were only three of them: ‘…*you get three takes* [i.e., contributors]…*and I wonder whether it is slightly delimited*…’ (Parent 1).
Subtheme 2.3 – Perspectives and representation	Include a wider range of perspectives (e.g. clips featuring parents) and better represent the social, cultural and ethnic diversity of LGBTQ+ youth: ‘…*seeing the same faces* [in it], *which is nice, albeit we need more of those brown faces*’ (Youth Worker 1).
**Theme 3: Functionality and development**	
Subtheme 3.1 – Improving the layout, format and navigation	Oneself needs refining in terms of its structure and presentation: ‘*Because again it's very click here, push that, what do you do* [next]?’ (Commissioner 2). It should also be suitably functional in a range of formats (e.g. on phones and devices).
Subtheme 3.2 – Choice and user options	It should provide differing content for a range of users (e.g. for 11–15 years old) and offer more options: ‘*So depending on what each person clicks it could also help you* [i.e., the developers of Oneself] *to target the resources*…’ (Youth Participant 10).
Subtheme 3.3 – Ideas for further evaluating Oneself	Suggestions were given about how the resource can be adequately evaluated or assessed in a timely fashion in real-world contexts. For example, ‘…*reach out to some secondary schools… see what they think about it…*’ (Youth Participant 11).
Subtheme 3.4 – Need for ongoing testing, refining and updating	Continue to test the resource (e.g. fix bugs and address navigational issues) as these occur and ensure Oneself is frequently revised: ‘…*make sure that you’re keeping everything up to date*’ (Commissioner 1).
**Theme 4: Safety and privacy**	
Subtheme 4.1 – Concerns and debates	Adult concerns or uncertainties alongside conflicting views amongst participants about the advice that should be given: ‘…*what worried me is that if you listen to them* [i.e., contributors]…*it comes across, coming out wasn’t so bad*…’ (Commissioner 2).
Subtheme 4.2 – Safety and risk management	Oneself should not ‘out’ anyone, participants also queried how data could be used: ‘…*online maybe you don’t exactly want people to know…you don’t want to put in too much information about yourself* [on the internet]….’ (Youth Participant 9).
Subtheme 4.3 – Harsh world warnings for LGBTQ+ youth	Young people should be made aware of hostilities (e.g. forms of abuse) directed towards LGBTQ+ people to allow them to form realistic expectations, and this is ‘…*much more significant than just saying, give your parents time*’ (Youth Worker 3).
**Theme 5: Reaching the end users**	
Subtheme 5.1 – Identifying the target users and their needs	Secondary school-aged youth seen as the primary end users, but Oneself should also be for adults, e.g. ‘*It's not specifically targeted for just queer people; it could also be about parents who want to become better allies and stuff*’ (Youth Participant 1).
Subtheme 5.2 – Promoting Oneself in a youth-friendly way	Practical ways to promote Oneself identified, including by using certain social media platforms to reach potential youths: ‘*I think a lot of younger young people do use TikTok…but then older ones will be using Instagram a lot more*’ (Youth Participant 10).
Subtheme 5.3 – Make it free and now	Ensure its freely available: ‘*I like that it's free, it's easily accessible to anyone who needs it because a lot of resources, especially with ones that tend to be more useful, have some of their content only accessible behind a pay-wall*’ (Youth Participant 11).
Subtheme 5.4 – Trustworthiness	Difficulties determining what is safe online and know what/who to trust: ‘*You know, this feels by its approach and I think the fact that it's got The Open University on there* [i.e., a university's logo on it]…*it feels like a trusted site*’ (Commissioner 1).
Subtheme 5.5 – Reaching the underserved	Libraries, youth centres and schools are valuable ways to reach youths but: ‘*…what you probably won’t get are the kids who haven’t actually come out…I wonder if maybe, probably trying to get it into…schools would be a useful* [approach]…’ (Parent 1).
**Theme 6: Mechanisms of impact**	
Subtheme 6.1 – Exploring your identity	Making sense of one's sexuality and/or gender is an individual journey and experiences vary: ‘*I like how it talks about how you don’t have to come out, it's not a forced thing, you can decide whether or not you want to*’ (Youth Participant 1).
Subtheme 6.2 – Sharing lived experience	It is validating to hear about the experiences of others, e.g. ‘*I think it was good to show that like it's not always pretty, like* [a] *great experience* [being LGBTQ+], *sometimes it can be really hard and difficult…people can get through it*…’ (Youth Participant 6).
Subtheme 6.3 – Informativeness and quality	The resource was generally seen as informative and it included useful wellness-orientated content (e.g. relaxation exercises as well as ‘…*downloads which are very informative*...’, Youth Worker 6).
Subtheme 6.4 – Balance needed in key messaging and advice	Positive messaging which attempts to ‘…*increase optimism*…’ (Youth Participant 9) needs to be balanced with realism (e.g. when coming out): ‘…*a lot of the people that I know who aren't out…definitely have a lot of anxiety about it*…’ (Youth Participant 9).
Subtheme 6.5 – Need to go further and delve deeper	Oneself requires additional content (e.g. ‘…*maybe have like frequently asked questions*…’ Youth Worker 1) and more real-life scenarios should be explored in-depth (e.g. managing life within an unsupportive family) including in the audio-visual content.
Subtheme 6.6 – How support networks can be used	Oneself could be used individually, and with families and/or with professionals, as LGBTQ+ youth will likely require the support of others therefore ‘…[Oneself should further] *link to trusted LGBTQ+ websites*’ (Commissioner 1).

*Clarity and accessibility*. Participants recommended that the language in Oneself be made clearer and more accessible. Specific words or phrases were also highlighted as needing adjustments, such that the suitability of ‘chilling out’ as a title to accurately encapsulate the relaxation and self-care section of Oneself was queried, as ‘chilling out’ implied something else to some participants. Certain elements needed written text, especially sub-titles/closed captions throughout all of the audio-visual clips. By contrast, some participants suggested an audio format instead of written text for certain elements:

*Instead of having it as words, one would have it as an audio, so then imagine a young person putting on headphones and they play and they can close their eyes and they can start to follow that voice that's speaking …* (Youth Worker 7)

There were different opinions on the wordiness of Oneself, such that one participant stated: ‘*Like I just looked at that first line and went, I’m not reading all that*’ (Young Person 3, Session 1) and later ‘…*less reading and more experiencing*’ (Young Person 3, Session 2), conversely an adult participant concluded that ‘…*there's not too much wordy content*’ (Parent 2). Also, ensuring that the resource was accessible to those who have additional learning needs was deemed valuable, such as by ‘…*making the font bigger*’ (Young Person 1, Session 3) and by improving colour contrasts.

*Appeal and depictions*. Perspectives differed in relation to the appeal of the resource and the relatability and representativeness of it. Oneself's colour scheme and overall presentation were perceived favourably. On the contrary, there were aspects that were off-putting. Having relatable ‘*real life experience*’ (Parent 1) to draw upon was viewed positively, but the older ages of, and small number of contributors were limitations:

*I think the only thing I would say overall, the three people you’ve got that do the videos …I wonder on some of the clips around school and things like that, are they young enough to be truly reflective of our school age young people?* (Commissioner 1)

Participants also noted that Oneself should include a wider range of perspectives and seek to better represent the diversity of sexual and gender minoritised adolescents, in line with expectations relevant to the local population (e.g. England). For instance, representation from a South Asian person was noted as absent:

…*the video representations are two white people* [i.e., Chloe and Lilly] *and one black person* [i.e., Georgie], *but across it… I haven’t seen a picture or a video of someone who is South Asian*… (Youth Worker 1)

*Functionality and development*. Participants described some issues and made suggestions about the layout, format and presentation of the resource. For instance, but somewhat in conflict with another recommendation (cited under Subtheme 1.4 – Accessibility by a young person), Young Person 8 said: ‘*I think just from a formatting point of view, sometimes I have to like scroll a bit to see all of it and I think if it was a little bit smaller, it would be quite nice*’ (Session 1). Participants also recommended the resource be functional in a range of formats. Further choice and user options were proposed (e.g. an online chat function). These options also included providing differing content depending on the age of the user. This was considered important, as reinforced by Commissioner 2, who stated: ‘…*is a 12-year-old the same as a 19-year-old? Absolutely not, one is a legal adult 18 plus, the other is a child and there's a big difference*’.

A range of suggestions were offered about how the resource could be adequately evaluated or assessed in real-world contexts. Participants also noted that Oneself would need ongoing testing, refining and updating: ‘*I also think it's important to, for it to be forever changing, forever progressing as well*’ (Youth Worker 1). Technical issues were encountered during sessions with young people, such that several instances of ‘*bugging out*’ (Young Person 6) that required attention were identified by most of the adolescents.

*Safety and privacy*. Participants were mindful of the unique challenges and contexts that sexual and gender minoritised adolescents contend with, and they discussed a range of safety and privacy considerations, as these applied to Oneself. Adult participants expressed concerns or uncertainties, for example around the issues inherent in pursuing the idea of a possible chat function in the future (suggested under Subtheme 3.2 – Choice and user options) which was considered: ‘…*hugely problematic and needs to be staffed and safeguarded*’ (Youth Worker 3). One adult participant (i.e. Commissioner 2) thought that the resource needed to be ‘…*balanced and truly reflective of current government guidelines* [on Relationships and Sex education/RSE]’. However, the same participant noted: ‘*The frustration at the moment is there's information about what should be taught* [in schools]*, but there's no guidance on how it should be taught and what resources you can use…*’ and it was acknowledged by the participant that government guidance on RSE in secondary schools (will again) be reviewed. Participants highlighted that Oneself should not ‘out’ users, and that it should include safety features (e.g. a quick escape button). Participants also reinforced that their user data needed to be secure. Adults and young people thought that sexual and gender minoritised adolescents should be made aware of the hostilities (e.g. being made homeless after coming out) and the general ambivalence of wider society towards them, and that Oneself should reflect this. As Youth Worker 5 commented (also linked to Subtheme 2.2 – Relatability and realness): ‘*Initially like I said, if you can find that brave person whose coming out didn’t go so well, was rejected, but since then they’ve accepted themselves and are okay, maybe they had to cut off their family*’.

*Reaching the end users*. Steps were considered by participants about how Oneself could get to the users who required it. Secondary school-aged adolescents were broadly seen as the primary end users, but their needs vary and segmentation by age group was suggested (see also Subtheme 3.2 – Choice and user options). It was also recommended that the resource be expanded for users beyond young people, including for parents. This was suggested by Parents 1, 2 and 3, Youth Workers 4, 8 and 9. Participants suggested Oneself be promoted in a youth-friendly way to reach the adolescents that needed it, in particular, it was recognised that social media is both beneficial and ubiquitous for LGBTQ+ people. The need to be ‘*out there*’ (Parent 3, Youth Workers 4 and 7) was reinforced by participants, as was the requirement that it be free.

The trustworthiness of Oneself, and all digital health resources, was seen as an important consideration: ‘*I think for everything nowadays, you do a dreadful Google search don’t you and it's what is safe, what isn’t safe?*’ (Commissioner 1). However, the same participant provided a warning related to reaching the end users: ‘*I would just say be prepared just for a little bit of stupid backlash from ill-informed narrow-minded people. But that's only because I’ve had a resource* [developed specifically for LGBTQ+ people] *that we’ve had to take down because of those things…*’. It was acknowledged that accessing those yet to ‘come out’ would be difficult, with schools a useful means to reach them.

*Mechanisms of impact*. There were a range of ways in which participants could see Oneself having a positive impact. It was observed that making sense of an adolescent's sexuality and/or gender was a dynamic process, but that this process could usefully be supported in the resource: ‘…*you can start your journey out maybe being bisexual for example, and as you grow older and exploring yourself you may feel, it's not this hat that I fit in…It can change and evolve, and I think that in itself could be seen, in quotes* [i.e., even more explicitly within Oneself]’ (Youth Worker 4). Moreover, the resource supporting choice or agency in one's life was recognised as valuable. The strengths of lived experience, especially personal anecdotes being shared with Oneself users, were viewed favourably by adolescents. As Young Person 3 explained: ‘*They* [i.e., Lilly, Chloe and Georgie] *just look like they were all happy in themselves and they were dressing how they were comfortable with and that's really good and important for young people to see*’. The broad consensus was that the resource was a high-quality intervention (e.g. ‘*I think it's really good content*’ – Young Person 1), that it was educational (e.g. ‘*I liked how informative it was*’ – Young Person 10) and that it contained useful wellness-orientated content. Participants noted that the key positive messaging needed to be balanced with realism (e.g. making sense of a person's sexuality and/or gender can be difficult), which was also related to Subtheme 4.3 – Harsh world warnings for LGBTQ+ youth. It was suggested that Oneself required more content (e.g. a full range of strategies to address bullying) and further real-life scenarios to more fully extend its possible impact. Finally, although Oneself was envisaged as being used in a variety of ways, alongside using this resource it was noted that sexual and gender minoritised adolescents will require the support of others (e.g. third sector organisations) to optimise Oneself's potential.

## Discussion

### Summary of findings

We conducted a formative evaluation of a digital intervention co-created with sexual and gender minoritised adolescents. The results of the SUS and the satisfaction survey suggest that Oneself was well received. For example, the mean participants’ SUS score was 78.8%, which corresponds to a ‘B+’ grade (on the Sauro–Lewis ‘A+’ to ‘F’ curved grading scale).^27^ Although the current formative evaluation was not designed or powered to test preliminary effectiveness, mean scores on the WHO-5 Wellbeing Index, immediately before the first think aloud session and immediately after the last think aloud session, increased from 51.6% to 54.7%. This improvement was recorded after adolescent testers used Oneself for a mean time of less than 2 hours over a period of three weeks, whilst engaged in think aloud sessions. Adolescents, and 13 of the 14 adults, thought that Oneself would appeal to LGBTQ+ young people. Furthermore, all but two participants (both adults) reported that they would recommend it to other LGBTQ+ young people.

### Comparisons to previous literature

Although the consensus was that Oneself was appealing and almost all the participants would recommend the resource to others, certain challenges remain. In common with other digital mental health tools interventions, most of these interventions are not available to young people outside of a research context.^14^ An exception is SPARX which is available in specific geographical locations. However, prior research suggests that a ‘mainstream’ intervention like SPARX, as delivered in New Zealand, may be less effective for gender minoritised adolescents.^32^ Steps have been taken to refine SPARX, in response to sexual and gender minoritised young peoples’ feedback,^33^ but sexual and gender minoritised adolescents in England had previously highlighted that a British-orientated bespoke LGBTQ+ specific digital resource is required.^15^ For example, sexual and gender minoritised adolescent participants in England wanted a more explicit focus on them and their needs, and not a modified version of SPARX or Rainbow SPARX.^15^ Additionally, when reviewing Rainbow SPARX, some participants in our earlier study indicated that the spoken dialogue, due to the ‘Kiwi’ accents, was at times difficult to understand.^15^

The socio-political context is also a noteworthy problem, as Commissioner 1 in their interview recounted how they had developed an intervention for LGBTQ+ young people they ‘…*had to take down*…’. The overall milieu towards sexual and gender minoritised adolescents has already been described as a key issue in the field, with McDermott et al.^34^ stating, about the UK context, that ‘…to support LGBTQ+ youth mental health is to understand that they live in a cis-heteronormative [i.e., where being cisgender and heterosexual is preferred, normal and healthy] world that, despite improvements, continues to either explicitly denigrate LGBTQ+ identities or marginalise and silence those lives’ (pp. 116–117). Participants wanted Oneself to be made available, after additional refinements had been made, but securing future research funding is challenging. Potential difficulties obtaining funding could reflect the organisational inertia, lack of prioritisation and/or systemic stigma towards LGBTQ+ young people.^2^ This is particularly concerning given their increased risk for mental ill-health^4,5^ and the need to counteract minority stress.^3^

We initially planned to use dramatisations within Oneself, but preliminary co-design work reinforced a strong preference for authentic real-life experiences instead.^19^ Consequently, we used three contributors who shared their lived experience. However, a challenge persists around how to be evidence-based or scientific and ‘*balanced*’ (Commissioner 2) alongside integrating this with diverse lived experience.^35^

### Strengths and limitations

The study has limitations, such as the relatively small sample size and exploratory nature of the research, which limits generalisability. The samples were drawn from England rather than the whole UK, which is also a limitation. Moreover, the adolescent sample was approximately three-quarters White, however gender minoritised participants were particularly well represented. Specifically, in the adolescent sample, eight (72.7%) stated that their gender was different to that they were assigned at birth. This is also potentially a limitation, in that the views of cisgender LGBTQ+ young people were under-represented in this study.

The study has strengths, such that we published a protocol at the start of the work^18^ and we outlined notable changes (e.g. that we did not use dramatisations as initially intended). Another strength is that we provided a detailed account of our co-design processes,^19^ when it has been estimated that only half of the interventions created for LGBTQ+ populations engage LGBTQ+ communities in their design processes.^16^ Finally, we drew on the evidence-base when developing Oneself, by conducting a scoping review of the relevant literature^17^ and using this research to support the content development of the intervention.

### Future research and challenges

Oneself demonstrates that a promising intervention can be made for under £50,000 (approximately US$61,000) in direct costs.^19^ But issues remain, such that adults and adolescents described the need for ongoing developments and refinements for LGBTQ+ youth mental health interventions, such as Oneself. The implications are that resourcing will be required for ongoing costs, but securing longer-term funding is challenging, and this is a notable and urgent concern for a range of digital health intervention developers and researchers. We are exploring a range of options including how to ensure Oneself would get to those who would benefit the most from it, as we remain mindful that interventions often miss those who really need them.^36^ This includes sexual and gender minoritised adolescents currently in the most challenging of environments, not because they are hard to reach (as seen with underserved populations generally), but they are easy to neglect.^36^

## Conclusions

This paper described the formative evaluation of a digital intervention to promote the mental well-being of sexual and gender minoritised youth. Our study found that participants using Oneself thought that it was appropriate for LGBTQ+ adolescents. They also indicated that it was suitable for their non-LGBTQ+ adolescent peers as well as adult allies. Some participants indicated that this intervention may be well placed to reach or affect the lives of adolescents who are not yet ‘out’, and those who are unsure and/or questioning. Quantitative results representing well-being, usability and satisfaction findings were mainly positive. For example, users described in positive terms Oneself's key mechanisms of action for promoting well-being, which included advice about coming out (or not coming out). There was suggestion of an urgency to make such interventions available freely and immediately, particularly for the adolescents who are often overlooked. But research funding for projects aimed to improve the lives of LGBTQ+ people in the UK is limited, and there has been a dearth of mental health interventions designed for LGBTQ+ young people in the UK and elsewhere. This threatens progress in relation to digital health interventions and initiatives such as Oneself.
